# Is the mitochondrion a promising drug target in trypanosomatids?

**DOI:** 10.1590/0074-02760210379

**Published:** 2022-02-21

**Authors:** Yasmin Pedra-Rezende, Ana Cristina Souza Bombaça, Rubem Figueiredo Sadok Menna-Barreto

**Affiliations:** 1 Fundação Oswaldo Cruz-Fiocruz, Instituto Oswaldo Cruz, Laboratório de Biologia Celular, Rio de Janeiro, RJ, Brasil

**Keywords:** trypanosomatids, mitochondrion, oxidative stress, chemotherapy, mitochondrial protein import, bioenergetics

## Abstract

The trypanosomatids *Trypanosoma brucei*, *Trypanosoma cruzi* and *Leishmania* spp. are etiological agents of important neglected tropical diseases, affecting millions of people worldwide, and the drugs available for these diseases present several limitations. Novel efficient and nontoxic drugs are necessary as an alternative to the current chemotherapy. The unique mitochondrion of trypanosomatids and its peculiar features turn this organelle a potential drug target. Several phenotypic studies describe the damage in the parasite mitochondrial ultrastructure, but the molecular target is unknown. Few reports demonstrated the electron transport system (ETS) as a target due to the high similarities to mammalian orthologues, hence ETS is not a good candidate for drug intervention. On the other hand, antioxidant enzymes, such as trypanothione reductase, and an alternative oxidase (AOX) seem to be interesting targets; however no high active inhibitors were developed up to now. Finally, due to the remarkable differences to mammalian machinery, together with the high biological importance for the parasite survival, the mitochondrial import system stands out as a very promising target in trypanosomatids. Archaic translocase of the outer membrane (ATOM) and translocase of the inner membrane (TIM) complexes, which mediate both protein and tRNA import, composed by specific subunits of these parasites, could be excellent candidates, deserving studies focused on the development of specific drugs.

The public health problem

The human African trypanosomiasis, Chagas disease and leishmaniasis are neglected tropical diseases caused by the protozoa parasites *Trypanosoma brucei*, *Trypanosoma cruzi* and *Leishmania* spp respectively. These illnesses affect millions of people worldwide with high morbidity and mortality rates and lead to critical social-economic implications, since they persist under conditions of poverty and are concentrated in impoverished populations in the developing world.^1^ Vaccines are not available, and the treatment of these diseases is based on drugs developed more than 50 years ago, especially due to the little or no prospect of financial gain, which discourages the search of new molecules by pharmaceutical industry. Additionally, current chemotherapy approaches have severe limitations, including high host toxicity, long-term treatments, and emergence of drug-resistant strains.^2^ ^,^ ^3^

Potential drug targets in trypanosomatid mitochondrion and oxidative stress generation

Parasites of Trypanosomatidae family exhibit the most typical eukaryotic organelles; however, some particular structures are also present, and have been pointed as potential targets for drug intervention. Unlike mammalian cells, trypanosomatids have a unique mitochondrion with several important metabolic peculiarities, which accredits this organelle as an excellent target for the development of novel therapeutics approaches.^4^ ^,^ ^5^ Such mitochondrion is also the most recurrent drug target described in mechanistic studies, with different classes of drugs inducing the organelle swelling, and impairing its matrix electron density and cristae ultrastructure (Fig. 1).^6^ ^,^ ^7^ ^,^ ^8^ ^,^ ^9^ Despite this promising scenario, mitochondrial phenotypes are commonly observed only by electron microscopy, with few reports assessing the real impact of these compounds in the organelle physiology.

**Fig. 1:**
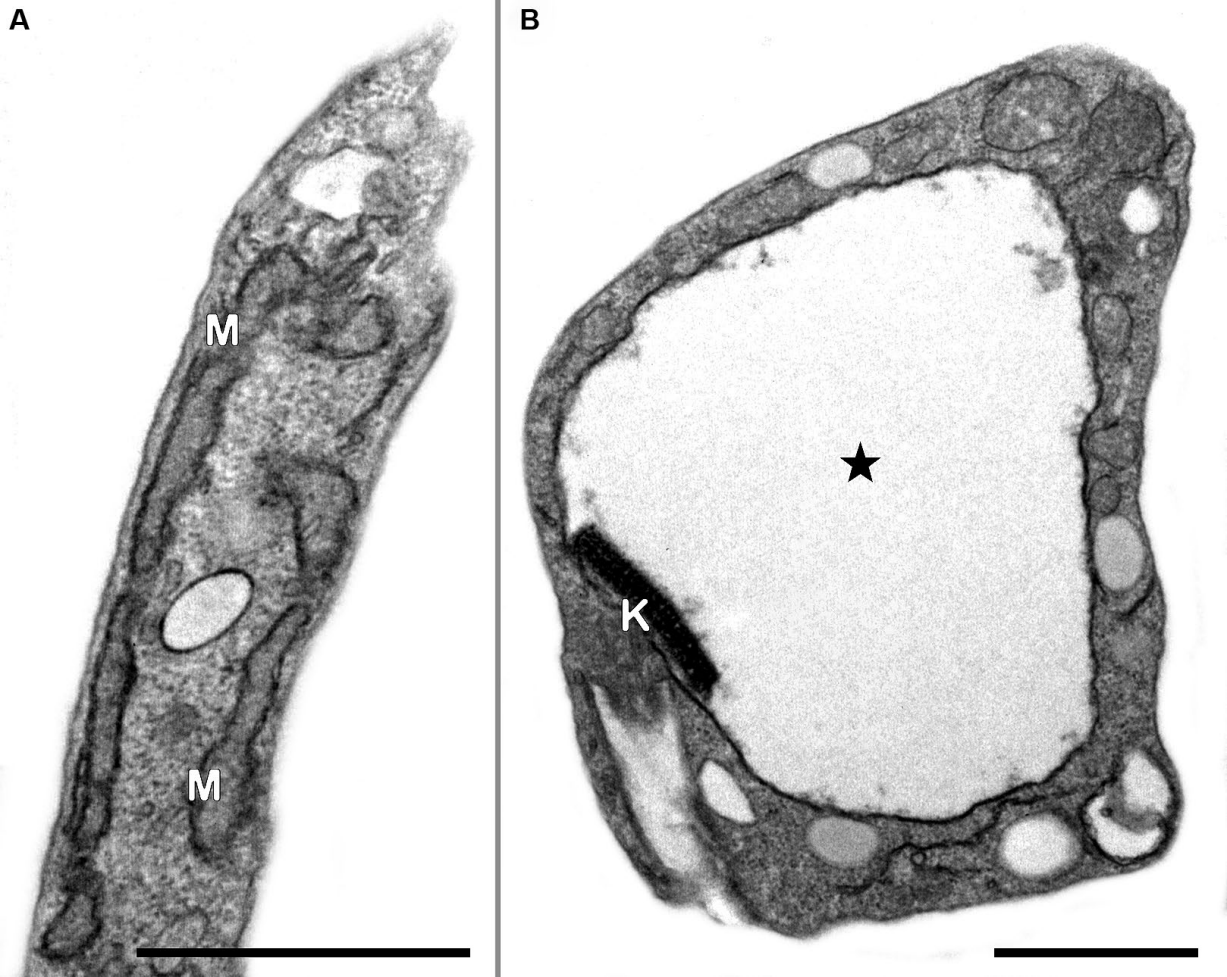
mitochondrial swelling is the most recurrent ultrastructural phenotype detected in trypanosomatids after the treatment with drugs. (A) Untreated parasite presenting typical elongated morphology of the mitochondrion (M). (B) Treated parasite showing a remarkable dilation of the organelle with loss of the cristae and electron density of the matrix (star). K: kinetoplast; Bars = 1 µm.

In general, mitochondria are organelles essential to aerobic organisms and compartmentalise fundamental physiologic processes. A cytochrome-independent alternative oxidase (AOX) in trypanosomatids, mainly found in long slender bloodstream form of *T. brucei*, is a promising drug target since this molecule is absent in mammalian cells.^10^ This parasite form relies entirely on the catabolism of glucose, an abundant energy source in the bloodstream of mammalian host, using AOX as terminal oxidase enzyme in the aerobic respiratory pathway. Thus, the nicotinamide adenine dinucleotide (NADH) formed during glycolysis is re-oxidised by O_2_ in the parasites’ mitochondrion in a process uncoupled to oxidative phosphorylation, and dependent of a mitochondrial membrane-associated glycerol-3-phosphate oxidase system, comprising a FAD-dependent glycerol-3-phosphate dehydrogenase, ubiquinone and AOX.^11^ Although, in bloodstream forms of *T. brucei*, the activity of mitochondrial complex IV (cytochrome oxidase) can be completely replaced by AOX, both molecules coexist in parasite’s procyclic form, a life cycle stage marked by strong upregulation of mitochondrial energy metabolism and with components constituting a complete cytochrome-containing electron transport system (ETS).^12^ ^,^ ^13^ Additionally, the presence of AOX in other pathogenic trypanosomatids was not confirmed, despite the occurrence of complex IV-independent oxygen consumption has been described in *T. cruzi* and *Leishmania (Leishmania) donovani*.^14^ ^,^ ^15^ Altogether, these data raise the question whether an inhibitor of AOX also could be a good candidate to the treatment of Chagas disease and leishmaniasis, since *T. cruzi* and *Leishmania* spp. have a more active mitochondrial metabolism, similarly to *T. brucei* procyclic form.

In addition to the pivotal role for energy production, mitochondrion also participates directly in the establishment of oxidative stress in eukaryotes, since the electron leakage from ETS leads to the partial reduction of oxygen and, consequently, the production of reactive oxygen species (ROS).^16^ In contrast to mammals, complex I (NADH-ubiquinone oxidoreductase) of trypanosomatids present low ROS production, which is directly associated with its truncated structure and low activity.^17^ ^,^ ^18^ ^,^ ^19^ Thus, the energy production in these parasites is fully dependent of complex II (succinate-ubiquinone oxidoreductase), and the succinate oxidation leads to electron transfer to the complex III (ubiquinolcytochrome c oxidoreductase) via ubiquinone.^19^ ^,^ ^20^ Although complexes II and IV are not common electron leakage sites, their inhibition by thenoyltrifluoroacetone (complex II inhibitor) or potassium cyanide (complex IV inhibitor) increases the ROS detection in trypanosomatids, as a consequence of electron flow interruption through ETS.^21^ ^,^ ^22^ ^,^ ^23^ Similar to mammalian cells, complex III and ubiquinone Q cycle are the major producers of ROS in these parasites. Such production occurs when ubiquinone remains for longer periods of time in its partially reduced form, due to the transference of only one electron from the complexes and formation of semiquinone, an intermediate extremely reactive and that can partially reduce other molecules present at the site, such as O_2_.^24^ ^,^ ^25^ In *T. cruzi* and *L. (L.) donovani*, the treatment with antimycin A (complex III inhibitor) increases ROS production in parasite stages found in the insect.^21^ ^,^ ^22^

Although basal production of ROS is crucial for cell physiology, and fluctuations in their levels can occur in response to certain stimuli, high concentrations of these molecules induce oxidative stress, being necessary the removal in order to avoid toxicity.^26^ ^,^ ^27^ It has been extensively described that chemical characteristics can confer high redox potential to some classes of drugs, resulting in ROS production during the treatment.^6^ ^,^ ^28^ ^,^ ^29^ ^,^ ^30^ ^,^ ^31^ ^,^ ^32^ ^,^ ^33^ The first mechanistic studies evaluating the effects of quinones in trypanosomatids were performed in the late 1970’s. In *T. cruzi*, epimastigotes treated with β-lapachone had increased mitochondrial ROS production, which was inhibited by the addition of the antioxidant enzyme superoxide dismutase. In this case, the generation of ROS was related to reduced respiratory rates and mitochondrial swelling in treated parasites.^34^ ^,^ ^35^ ^,^ ^36^

Previous studies of our group contributed to the elucidation of the trypanocidal mechanisms of β-lapachone derivatives. The reaction of this naphthoquinone with different aldehydes, in the presence of ammonium acetate, led to imidazole ring insertion, generating several naphthoimidazoles. N1, N2 and N3 (Fig. 2) were the most promising derivatives, presenting activity up to 18-fold higher on *T. cruzi* infective form than the reference drug.^37^ ^,^ ^38^ ^,^ ^39^ Despite the impairment of several cellular processes by these naphthoimidazoles, the most prominent effect was observed in the protozoa mitochondrion. Ultrastructural analysis pointed to this organelle swelling in epimastigotes and bloodstream trypomastigotes, a significant reduction of mitochondrial membrane potential (ΔΨm) as well as in the activity of complex III.^40^ ^,^ ^41^ Corroborating these findings, in 2019, our study showed that these three derivatives compromised mitochondrion metabolism, disrupting O_2_ consumption and complexes II-III and IV activities. In addition, ROS were directly associated with the trypanocidal effect, N2 and N3 decreasing the electron flux through ETS and increasing the mitochondrial ROS production. In contrast, ROS production by N1 did not result from mitochondrial damage, so it may be a consequence of the antioxidant system inhibition.^32^ More recently, the correlation between mitochondrial ROS production and trypanocidal effect of the naphthoimidazole N4 (Fig. 2) was also demonstrated. The activity of N4 involves elevated ROS production and decreased activity of complex II-III in the first hours of treatment (2 and 4 h); apart from the morphological and functional impairment of this organelle in longer times (24 h).^33^ In 2009, our group also evidenced the effect of naphthofuranquinones in the mitochondrial metabolism of *T. cruzi* stages. These compounds exhibited powerful effects on the protozoa mitochondrion, which appeared drastically swollen and with a washed-out matrix phenotype, in addition to significant reduction of complex I-III activity and succinate-induced O_2_ consumption. It was suggested that naphthofuranquinones could interfere with electron flow at the inner mitochondrial membrane, deviating electrons from the total ubiquinone pool, resulting in semiquinone formation and oxidative stress.^6^ In addition, the treatment of *T. cruzi* with 1,2-naphthoquinone and 9,10-phenanthrenquinone led to superoxide anion and hydroxyl production, corroborating redox stress as the main mechanism of quinones.^42^

**Fig. 2:**
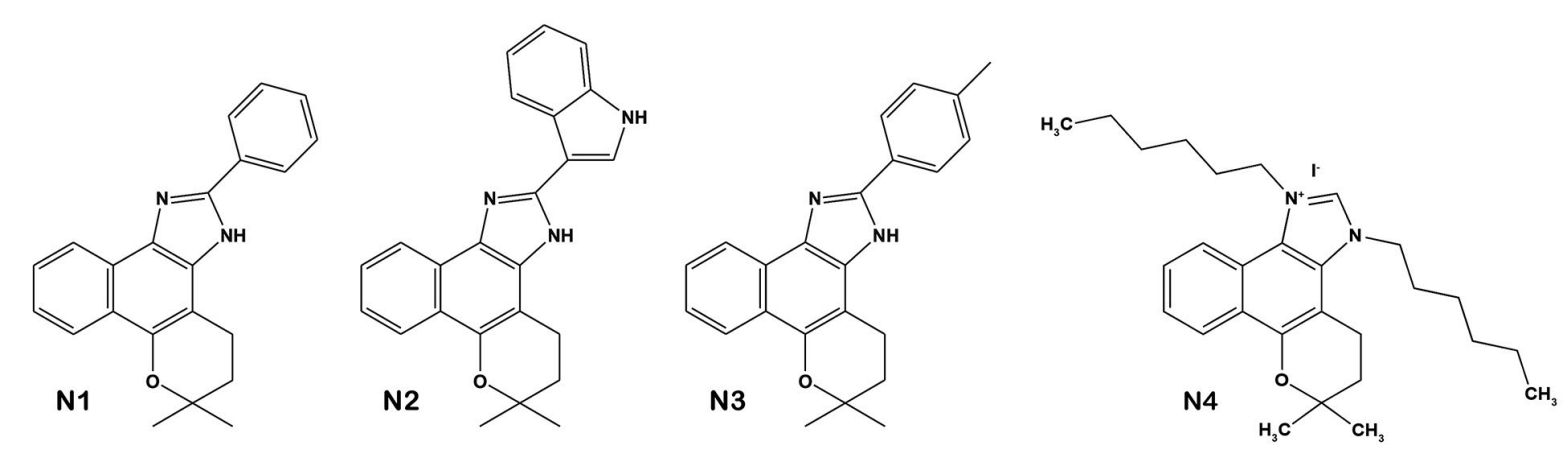
chemical structures of naphthoimidazoles N1, N2, N3 and N4.

Quinones also had their mechanism of action assessed in other pathogenic trypanosomatids.^43^ In *L. (L.) donovani*, a benzophenone-derived bisphosphonium salt targets complex II and causes dramatic mitochondrial injury, including organelle swelling, ΔΨm and O_2_ consumption decrease, as well as prominent impairment of cytoplasmic ATP levels^7^. Tafenoquine inhibits complex III, increases mitochondrial ROS production and elevates intracellular Ca^2+^ levels, inducing this organelle depolarisation in promastigotes forms of *Leishmania* spp.^30^ Similar findings were observed in *L. (L.) donovani* treated with clerodane diterpene, leading to cytoplasmic cytochrome c release and disruption of ATP production.^31^

For many years the idea that trypanosomatids were more sensitive to oxidative stress, due to the lacking of classical antioxidant defenses such as catalase and glutathione peroxidase, led to the search for novel ROS-producing compounds in these parasites.^44^ ^,^ ^45^ However, this hypothesis has been proved incorrect, since the parasites’ antioxidant system is extremely efficient fighting against reactive species derived from pharmacological intervention and/or produced in the host environment. Differently from mammalian cells, trypanosomatids possess a specific antioxidant system, based on glutathione analogue trypanothione [T(SH)_2_].^46^ T(SH)_2_ is the major redox reactive metabolite in these parasites, and participates in a wide range of enzymatic and non-enzymatic reactions, such as peroxide detoxification.^47^ ^,^ ^48^ Accordingly, T(SH)_2_ plays a key role in redox homeostasis in all trypanosomatids, supporting the infectivity and survival in the hosts, while the disruption of its metabolism could increase parasites´ susceptibility to oxidative stress. T(SH)_2_ pathway offers exceptional chances for the development of anti-trypanosomatids’ strategies, which is desirable to decrease drug development costs and to treat many related diseases.^49^ Despite the major efforts in this field, focusing on metabolic enzymes involved in T(SH)_2_ synthesis and regeneration, no promising inhibitor has been found up to now. It is interesting to note that trypanosomatids can survive with reductions of up to 90% in trypanothione reductase activity and T(SH)_2_ levels,^50^ ^,^ ^51^ making hard the development of drugs able to inhibit this pathway.

The mitochondrial protein import system is a promising drug target?

The unique mitochondrion of trypanosomatids is extremely dynamic, adapting its morphology and content in response to environmental conditions found in host system. The huge majority of mitochondrial proteins are encoded by nuclear genome, depending on translocase of the outer membrane (TOM), translocase of the inner membrane (TIM), sorting and assembly machinery (SAM) and oxidase assembly (OXA) complexes for the import of polypeptide sequences into the organelle (Table). The mitochondrial outer membrane (MOM) protein Tom40 is the usual entry of polypeptide sequences in the organelle of most eukaryotes; however, in trypanosomatids, Tom40 is absent and is replaced by a different import channel, termed ATOM (archaic translocase of the outer mitochondrial).^52^ This complex was first described in *T. brucei* and is formed by seven subunits, two of them (ATOM40 and ATOM14) with some homology to Tom40 and Tom22 present in yeast.^53^ Although the other subunits (ATOM69, ATOM46, ATOM19, ATOM12, and ATOM11) are exclusive of trypanosomatids, and have evolved independently of any subunit previously described in the TOM complex,^53^ ^,^ ^54^ there are functional similarities between mitochondrial import systems of these parasites and other eukaryotic cells. Except for ATOM46, all subunits are essential for the viability and mitochondrial protein import in trypanosomatids.^55^ SAM complex in MOM was also described in *T. brucei* and its core subunit Sam50 is highly conserved. As in other eukaryotes, trypanosome Sam50 mediates the biogenesis of β-barrel membrane proteins into the outer membrane.^56^ An orthologue of Sam35, a component of SAM complex in yeast, has been detected in the MOM of trypanosomes; however, whether it forms a complex with Sam50 is presently unknown.^55^ ^,^ ^57^

**TABLE Components:** of the mitochondrial protein import complexes in *Trypanosoma brucei*

Complexes	Subunits	Depletion effects	Yeast ortholog	References
ATOM	ATOM40	Reduction in levels of ATOM14, ATOM11, ATOM46, and ATOM69	Tom40	^52^ ^,^ ^53^ ^,^ ^82^ ^,^ ^83^
	ATOM14	Impairment of protein and tRNA import	Tom22	^53^ ^,^ ^83^
	ATOM46	not described	-	^53^
	ATOM69	Accumulation of cytosolic precursor proteins; parasite growth arrest	-	^53^
	ATOM11	Inhibition of tRNA import	-	^53^ ^,^ ^83^
	ATOM12	Inhibition of tRNA import	-	^53^
	ATOM19	Inhibition of mitochondrial protein import, ΔΨm and kDNA loss, decline in O_2_ uptake and parasite growth arrest	-	^84^ ^,^ ^85^
SAM	Sam50	not described	Sam50	^56^
	Sam35	not described	Sam35	^57^
OTHERS	pATOM36	Alteration in MOM protein composition, kDNA loss and increased distance basal body - MOM	-	^58^ ^,^ ^62^
	Erv1	Reduction in abundance of small TIMs and cysteine-rich substrates in MIS	Erv1	^70^ ^,^ ^71^
TIM	TbTim17	Inhibition of the mitochondrial import of proteins and the newly synthesised tRNA; impairment of the mitochondrial import of cytochrome oxidase subunit IV and decreased ΔΨm	Tim17/22/23	^54^ ^,^ ^75^ ^,^ ^76^ ^,^ ^86^
	TbTim62	Inhibition of import of mitochondrial proteins *in vitro*; reduction in mitochondrial protein content *in vivo*	-	^54^ ^,^ ^75^
	ACAD	not described	-	^54^ ^,^ ^75^
	TbTim42	not described	-	^54^
	TimRhom I	Impairment of cytochrome oxidase subunit 4 import	-	^54^ ^,^ ^87^
	TimRhom II	Impairment of cytochrome oxidase subunit 4 import	-	^54^ ^,^ ^87^
	TbTim50	kDNA overreplication, ΔΨm decrease and cell growth in the bloodstream form of *T. brucei*	-	^80^ ^,^ ^81^
	TbTim47	Inhibit *in vitro* import of mitochondrial proteins	-	^75^
	TbTim54	Inhibit *in vitro* import of mitochondrial proteins; impacts negatively the protein content *in vivo*	-	^75^
PAM	mHsp70	Impairs mitochondrial import of proteins and tRNA	mHsp70	^75^ ^,^ ^76^
	Mge1	not described	Mge1	^88^
	TbPam16	Parasite growth arrest	-	^79^
	TbPam18	Parasite growth arrest	-	^79^
	TbPam27	Parasite growth arrest; and accumulation of cytochrome oxidase subunit 4	-	^79^
Small Tims	Tim9	Parasite growth arrest and reduced levels of TbTim17	Tim9	^54^ ^,^ ^64^ ^,^ ^68^
	Tim10	Parasite growth arrest and reduced levels of TbTim17	Tim10	^54^ ^,^ ^64^ ^,^ ^68^
	Tim8/13	Parasite growth arrest and reduced levels of TbTim17	Tim8/13	^54^ ^,^ ^64^ ^,^ ^66^ ^,^ ^68^
	Tim11	not described	-	^54^ ^,^ ^66^
	Tim12	not described	-	^54^ ^,^ ^66^
	Tim13	not described	-	^54^

Trypanosomatids also possess a specific protein, named pATOM36 (peripheral ATOM36), which is associated with insertion of some proteins in mitochondrial membrane and mediates the assembly of MOM protein complexes. pATOM36 knockdown affects MOM protein composition by molecules that have classical α-helical transmembrane domains, including six subunits of the ATOM complex.^58^ ^,^ ^59^ ^,^ ^60^ Despite the similar function with the mitochondrial inner-membrane import machinery (MIM) complex of yeast, pATOM36 has different molecular weight and topology, and does not show any sequence similarity to Mim1 or Mim2 (subunits of MIM complex). Interestingly, the expression of pATOM36 in a MIM-depleted strain rescued the growth of the cells and to the assembly of TOM complex in yeast; in the same way, the expression of Mim1 and Mim2 was not enough to assembly of ATOM after *T. brucei* pATOM36 knockdown. Altogether, these data suggest that pATOM36 and MIM complexes are functional analogues, and that neither the trypanosomal molecule nor Mim1/Mim2 needs a specific partner protein to exert their function.^61^ pATOM36 also has a second function unrelated to mitochondrial protein import. It has been pointed to the specific localisation of this molecule at the tripartite attachment complex (TAC), a trypanosomatid structure that physically links mitochondrial genome (kDNA) to the basal body of the parasite.^59^ Thus, TAC function is to guaranteeing the perfect segregation of the replicated kDNA to both daughter cells after mitosis. The knockdown of *T. brucei* pATOM36 also resulted in the loss of kDNA and increased the distance between basal body and MOM, indicating that this molecule is essential to DNA inheritance.^62^ ^,^ ^63^

Small TIM (translocase of the inner mitochondrial membranes) proteins are a highly conserved family found in all eukaryotes. In *T. brucei*, six different small TIMs proteins with cysteine-rich motifs were described (TbTim9, TbTim10, TbTim11, TbTim12, TbTim13, and TbTim8/13); however, sequence comparisons do not allow the assignment of the trypanosomal TIMs to their counterparts in yeast and mammals.^55^ ^,^ ^64^ ^,^ ^65^ It has been determinated that all of these small TbTims are essential for *T. brucei* cell growth and are found tightly associated with TbTim17. Depletion of any of these severely hampers the stability of the TbTIM17 complex.^65^ ^,^ ^66^ ^,^ ^67^ ^,^ ^68^ The import of proteins in the mitochondrial intermembrane space (MIS) is mediated by MIA (mitochondrial intermembrane space assembly) system, consisting by Mia40 and Erv1 in yeast. Mia40 acts as receptor of newly imported cysteine-rich substrates and catalyses the formation of disulfide bounds, while the sulfhydryl oxidase Erv1 operates oxidising the reduced Mia40.^69^ Interestingly, although Erv1 and cysteine-rich substrates of the MIA system are present in trypanosomatids, there is no description of a Mia40 homologue encoded by parasites’ nuclear genome.^70^ ^,^ ^71^ Erv1 knockdown reduces the abundance of small TIMs and cysteine-rich substrates in MIS of *T. brucei*,^72^ pointing to the pivotal role of this orthologue. A recent study suggests the presence of another protein that may function as a Mia40 analogue. The depletion of Mic20, a thioredoxin-like protein member of the peripheral MICOS (mitochondrial contact site and cristae organising system) subcomplex, led to a decreased import of MIS proteins, including ETS assembly factors.^73^ Other components of peripheral MICOS subcomplex also were associated with mitochondrial protein import, such as Mic32 and Mic34.^74^

Unlike yeasts and mammals, trypanosomatids have a single TIM complex that imports and inserts all proteins in mitochondrial inner membrane and matrix. TIM complex is present in a high molecular weight complex and has only the subunit Tim17 homologue to TIM22 and TIM23 found in fungi and other eukaryotes.^63^ ^,^ ^75^ It has been described that downregulation of *T. brucei* Tim17 by RNAi strongly inhibits the mitochondrial import of proteins and newly synthesised tRNA,^76^ suggestive of the participation in the translocation of tRNAs across the mitochondrial inner membrane. Mass spectrometry analysis identified 20 proteins associated with the parasite Tim17. Knockdown of Tim47, Tim54 and Tim62 inhibited import of mitochondrial proteins *in vitro*, while the disruption of Tim54 and Tim62 also negatively impacts the protein content *in vivo*.^75^ ^,^ ^77^ The TIM subunits Tim42 and ACAD, an orthologue of a medium chain length acyl-CoA dehydrogenase, also were described.^54^ ^,^ ^75^ ^,^ ^77^ It has been described two rhomboid-like proteins, named TimRhom I and TimRhom II, associated with the presequence translocase of *T. brucei*, which are essential for growth and *in vivo* mitochondrial protein import.^54^ ^,^ ^55^ The presequence translocase needs an import motor, which in yeast is composed by presequence translocase-associated motor (PAM). The orthologues of the PAM subunits: Tim44, mHsp70, Mge1, Pam18 e Pam16 were found in *T. brucei* mitochondrion.^78^ Knockdown of mHsp70 reinforced the importance of this molecule for mitochondrial import of proteins and tRNA,^76^ while the downregulation of Pam18 and Pam16 affected the growth of procyclic but not of bloodstream stage of *T. brucei*. It also has been shown that the function of Pam18 has been replaced by TbPam27, which is specifically required for the import of mitochondrial presequence.^79^ A gene encoding a putative orthologue of yeast and mammal Tim50 was discovered in *T. brucei* It has been shown evidence that Tim50 phosphatase activity is involved in the regulation of proteins related to voltage-dependent anion channel (VDAC) in parasites’ mitochondrion. Since VDAC protein levels are dramatically altered according *T. brucei* life cycle stages, this data suggests that Tim50 may play a role in the regulation of mitochondrial activity.^80^ The expression of Tim50 was also described as essential for mitochondrial functionality, kDNA replication and cell cycle in bloodstream form of *T. brucei*.^81^ Despite that, some studies question the key role of Tim50 in these processes, since its disruption causes pleiotropic effects. Furthermore, Tim50 was not recovered in immunoprecipitations using tagged Tim subunits.^54^ ^,^ ^60^ The Fig. 3 and Table summarises all mitochondrial import machinery described in *T. brucei*.

**Fig. 3:**
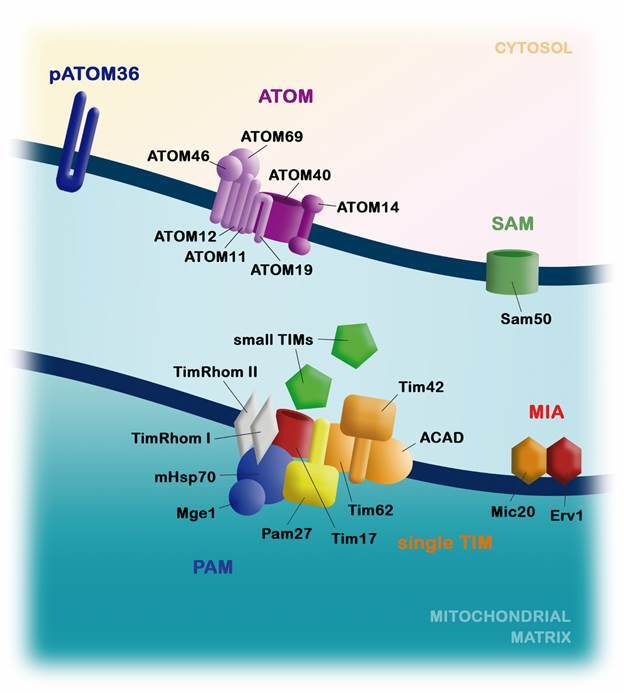
mitochondrial proteins import system in *Trypanosoma brucei*. As in other eukaryotes, trypanosomatids present translocase of the outer membrane (TOM), sorting and assembly machinery (SAM) and translocase of the inner membrane (TIM) complexes, composed by several subunits. Many differences between mammalian and protozoan import machinery can be detected. In trypanosomatids, ATOM40, ATOM14, ATOM69, ATOM46, ATOM19, ATOM12, ATOM11, Sam 50, pATOM36 are present in the outer membrane. Small TIMs and mitochondrial intermembrane space assembly (MIA) system are found in the intermembrane space. In the inner membrane, Tim17, Tim42, Tim62, ACAD, presequence translocase-associated motor (PAM) subunits (mHsp70 and Mge1) and TbPam27 are present. The presequence translocase is specifically related to TimRhom I and TimRhom II.

Concluding remarks

Among the mitochondrial functions, oxidative phosphorylation is one of the most important. The kDNA encodes a few protein-coding genes, many of which have to be edited to become functional mRNA. Thus, the mitochondrial requirements need to be supplied by proteins encoded in nuclear genome, including enzymes involved in biosynthetic and catabolic pathways, subunits of ETS complex as well as molecules related to redox homeostase maintenance. Despite the similarities between the mitochondrial protein import system of trypanosomatids and mammals, only few subunits of their machinery are conserved. Those differences reinforce the potential of this system as a drug target, allowing the development of more efficient and safe drugs against pathogenic trypanosomatids, since there is sufficient evidence that mitochondrial protein import is essential for parasite survival and infection. Furthermore, the identification and/or characterisation of mitochondrial import components in other pathogenic trypanosomatids are imperative.
